# Becker muscular dystrophy and successful intervention with mechanical thrombectomy of right atrial clot‐in‐transit with pulmonary embolism

**DOI:** 10.1002/ccr3.7390

**Published:** 2023-05-22

**Authors:** Mahfujul Z. Haque, Taha Akbar, Abdulmalik Saleem, Mashkur Husain

**Affiliations:** ^1^ Michigan State University College of Human Medicine Grand Rapids Michigan USA; ^2^ The Ohio State University College of Arts and Sciences Columbus Ohio USA; ^3^ Department of Internal Medicine Henry Ford Hospital Detroit Michigan USA; ^4^ Downriver Heart & Vascular Specialists PC Southgate Michigan USA

**Keywords:** Becker muscular dystrophy, mechanical thrombectomy, pulmonary embolism, right atrial clot‐in‐transit

## Abstract

This case report discusses the effectiveness of the Inari FlowTriever system in treating a right atrial (RA) clot in‐transit in a 55‐year‐old male patient with Becker's muscular dystrophy (BMD). BMD is an X‐linked recessive muscle disease caused by mutations in the gene that code for the protein dystrophin, which is associated with partially functional dystrophin in variable amounts. Right heart thrombi (RHT) are thrombi that can be visualized in the right atrium, right ventricle, or proximal surrounding vasculature. The Inari FlowTriever system was used to treat RA clot in‐transit and removed acute, subacute, and chronic clot in a single session without the use of thrombolytics and subsequent ICU stay. The estimated blood loss with the FlowSaver system was approximately 150 mL. This report complements the FLARE study by highlighting the effectiveness of the FlowTriever system for mechanical thrombectomy of RA clot‐in‐transit in a patient with BMD.

## Graphical abstract

This report discusses the use of the Inari FlowTriever system in treating a right atrial clot in‐transit in a patient with Becker's muscular dystrophy. Right heart thrombi are thrombi that can be visualized in the right atrium, right ventricle, or proximal surrounding vasculature. The Inari FlowTriever system was used to treat RA clot in‐transit and removed acute, subacute, and chronic clots in a single session without the use of thrombolytics and subsequent ICU stay.

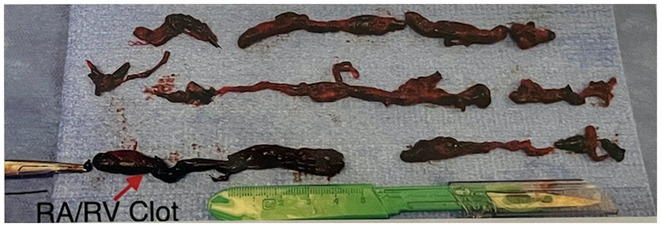

## 1 INTRODUCTION

Becker's muscular dystrophy (BMD) is a muscle disease with an X‐linked inheritance and is caused by mutations in the gene that code for the protein dystrophin. BMD is associated with partially functional dystrophin in variable amounts.^1^, ^2^ Patients with BMD may present with cardiac complications, specifically cardiomyopathy.^3^ These patients may also present with early right ventricular dysfunction and late‐onset left ventricle complications.^3^

Right heart thrombi (RHT) are thrombi that may be found in the right atrium (RA), right ventricle (RV), or proximal surrounding vasculature. These can feature different characteristics of being mobile or immobile.^4^ They may also adhere to devices in the body such as pacemakers.^4^ The association between the occurrence of right atrial (RA) clot in‐transit with subsequent pulmonary embolism (PE) ranges from nearly 4% to 18%.^5^ The mortality of cases without therapy is between 80% and 100%.^6^

Inari developed the FlowTriever system and intended for the treatment of pulmonary embolism through the peripheral vasculature without thrombolytics, as well as treating clots in‐transit in the right atrium. The device consists of a large lumen catheter and large bore syringe designed to rapidly extract large volumes of clot while mitigating blood loss. The catheter features three nitinol mesh disks in varying sizes depending on the size of clot. These disks are responsible for delivering the clot to the catheter for extraction (Inari Medical Inc). A recent case report details the effectiveness of FlowTriever device in treating PE noted by the rapid normalization of pulmonary artery (PA) pressure and SpO_2_.^7^ However, more extensive studies are required before this device is used as a main tool to treat acute PE.^7^ Our case report demonstrates the use of Inari FlowTriever to treat RA clot in‐transit in a patient with BMD. To our knowledge, this is the first report regarding successful mechanical thrombectomy (MT) in a patient with BMD.

## 2 CASE REPORT

**FIGURE 1:**
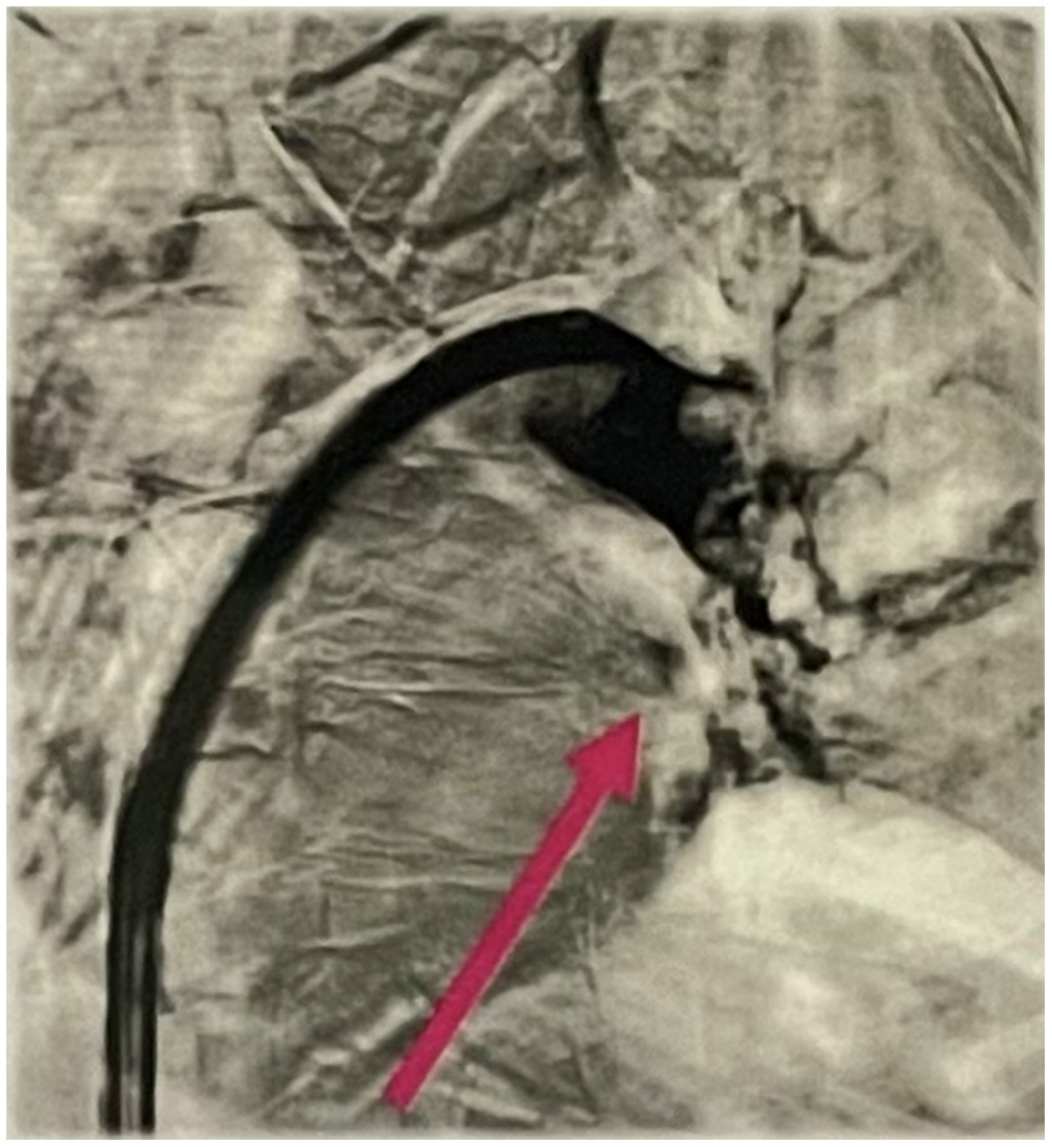
Occluded pulmonary artery Pre‐FlowTriever mechanical thrombectomy.

**FIGURE 2:**
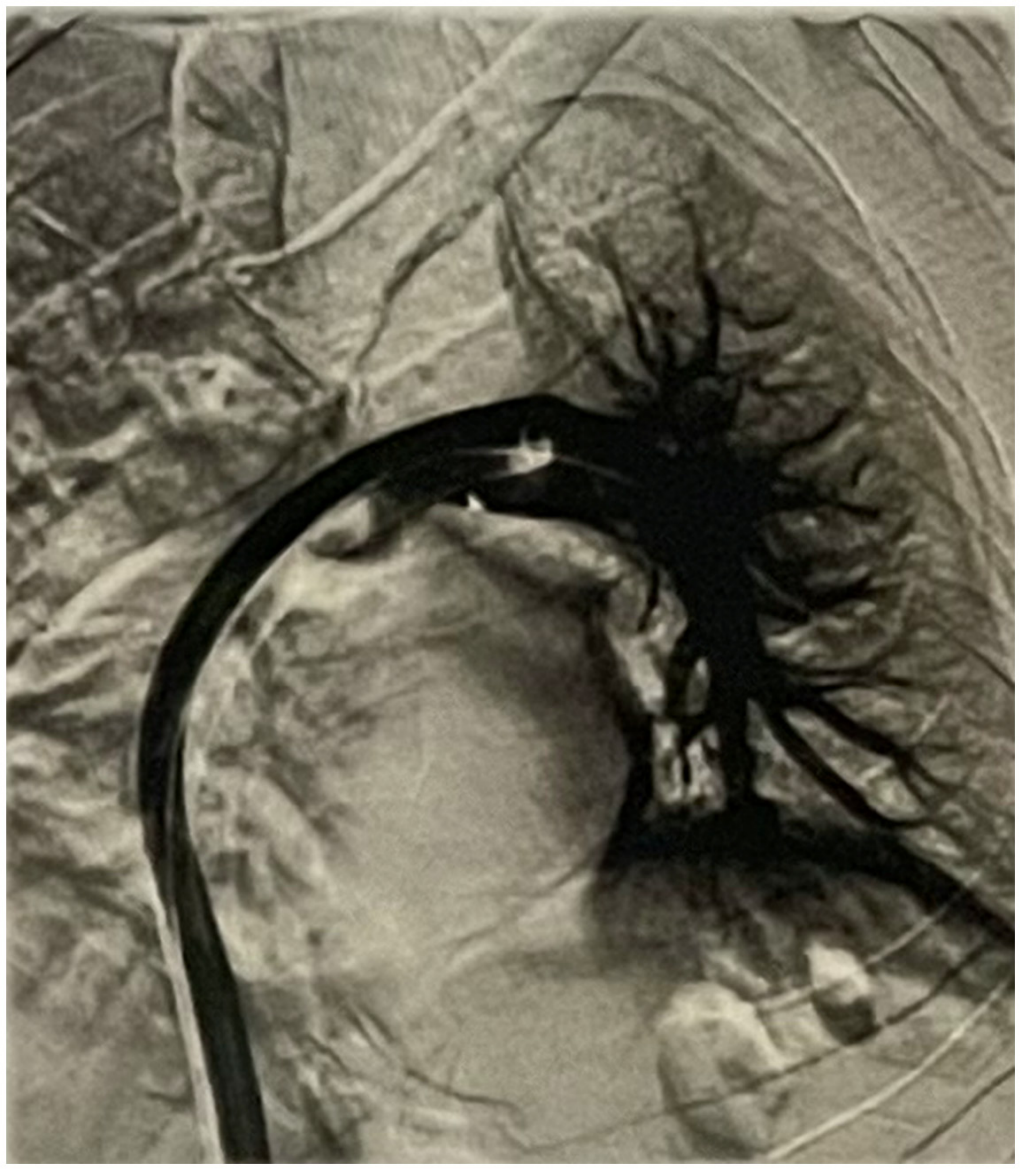
Clot removed Post‐FlowTriever mechanical thrombectomy.

**FIGURE 3:**
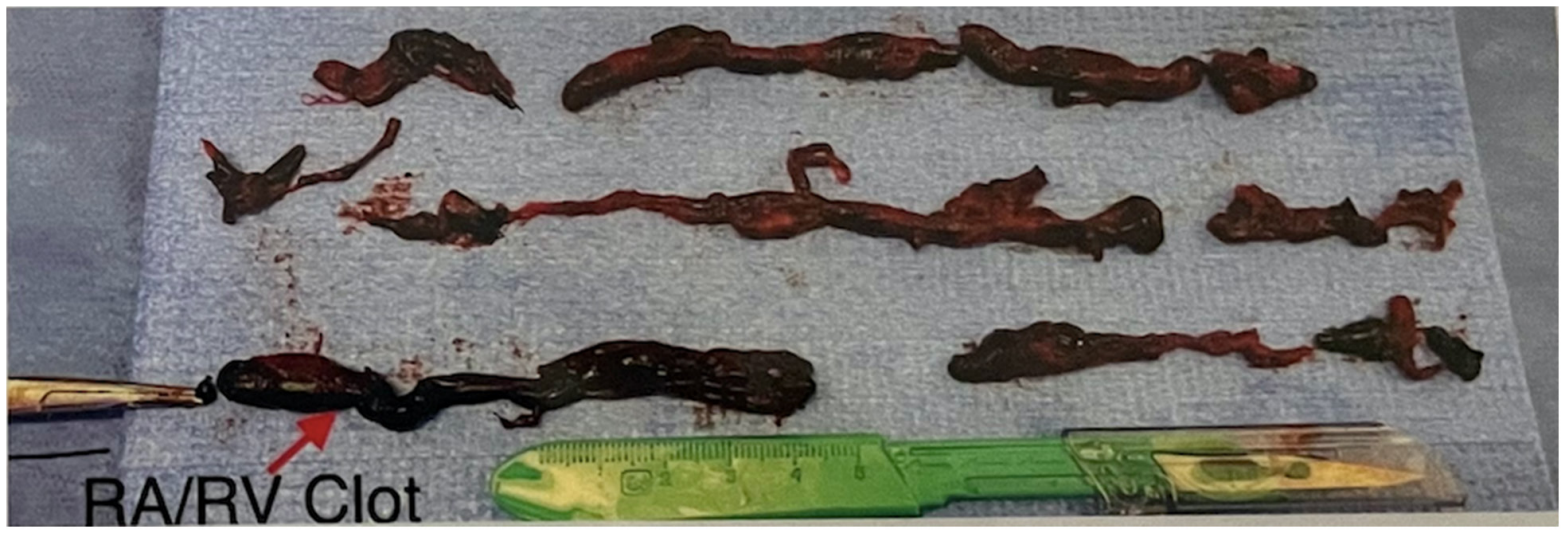
Gross image of removed clots.

A 55‐year‐old man with a history of Becker's muscular dystrophy (BMD) presented with a chief complaint of progressive dyspnea. The patient was hypotensive. The patient denied changes in mental status, loss of consciousness, and cold extremities. The patient was not taking any medications currently, for his dyspnea or BMD, and did not report any other pertinent findings during review of systems. The patient denies any history of smoking or use of corticosteroids. The patient denied taking hypotensive‐inducing medications including angiotensin‐converting enzyme inhibitors. Transthoracic echocardiogram revealed right ventricle enlargement and right atrial clot‐in‐transit extending through the tricuspid valve into the right ventricle. Computed‐tomography angiography (CTA) revealed bilateral pulmonary embolism. Figure 1 shows the occluded pulmonary artery pre‐intervention. In addition, the right ventricle/left ventricle ratio was 1.5. The venous doppler ultrasound confirmed bilateral lower extremity iliofemoral deep vein thrombosis. The patient was transported to the catheterization lab immediately for mechanical thrombectomy using the Inari FlowTriever and FlowSaver Blood Return system. The patient's pre‐intervention vitals were a heart rate of 105 beats per minute, SpO_2_ of 85%, respiratory rate of 28 respirations per minute, blood pressure of 92/61 mm Hg, and pulmonary artery pressure of 70/24 (37) mm Hg. The patient's pulmonary embolism severity index (PESI) was 115 points and classified Class IV. This is considered high risk with a 4.0%–11.4% 30‐day mortality in this group. The FlowTriever system removed acute, subacute, and chronic clots in a single session. Figures 2 and 3 displays the post‐intervention results and the removed clots. The post‐intervention vitals were a heart rate of 90 beats per minute, respiratory rate of 16 respirations per minute, blood pressure of 125/75 mm Hg, SpO_2_ of 95%, and pulmonary artery pressure of 29/14 (17) mm Hg. The patient was not given thrombolytics and did not have any ICU stay. The estimated blood loss with the FlowSaver system was approximately 150 mL. The patient reported complete resolution of dyspnea. Long‐term anticoagulation was prescribed to the patient to minimize the chances for recurrence.

## 3 DISCUSSION

Research regarding the cardiac involvement of BMD is limited. Although the patient did display a RV/LV ratio of 1.5, it was not possible to determine whether this is an acute process or due to the BMD. The role of the dystrophy is thus unclear in the patient's presenting symptoms. Although there are no standardized guidelines for the optimal treatment of acute PE,^8^, ^9^ available treatments include surgical embolectomy, systemic pharmacologic thrombolysis, and percutaneous vacuum‐assisted thrombectomy, in addition to conventional use of anticoagulation.^10^

The FlowTriever system is unique in that it is the only FDA approved catheter device for PE. The ability to maneuver into the pulmonary segmental branches and aspirate central and more distal clotting is a major advantage of this device. Additionally, a coaxial system allows for the use of contrast injections to track the progress of the procedure. A previous study known as the FLARE study showed the safety and effectiveness of the FlowTriever system with acute intermediate‐risk PE.^11^ The study reported significant improvement in RV/LV ratio and minimal major blood loss.^11^ The advantages suggested by the study included immediate thrombus removal, absence of thrombolytic complications, and a reduced need for post‐procedural critical care.^11^ However, there are some reports of complications with the use of this device.^12^ There are risks for pericardial effusion, tamponade, and paradoxical embolism.^12^ Additionally, there are reported clot displacements during the procedure and reported mortality due to prolongation of cardiogenic shock.^12^ This report complements the FLARE study by highlighting the effectiveness of the FlowTriever system for MT of RA clot‐in‐transit.^11^ The FlowTriever MT removed acute, subacute, and chronic clot in a single session without the use of thrombolytics and subsequent ICU stay.

## 4 CONCLUSION

The patient reported a complete resolution of their dyspnea and a notable improvement in their SpO_2_ and PA pressure following mechanical thrombectomy. This case report adds valuable information regarding the use of FlowTriever MT as an effective option for removal of RA clot‐in‐transit with pulmonary embolism in a patient with BMD.

## AUTHOR CONTRIBUTIONS

**Mahfujul Z. Haque:** Conceptualization; data curation; formal analysis; investigation; methodology; project administration; supervision; validation; visualization; writing – original draft; writing – review and editing. **Taha Akbar:** Conceptualization; formal analysis; methodology; resources; writing – original draft. **Abdulmalik Saleem:** Conceptualization; data curation; writing – original draft. **Mashkur Husain:** Investigation; methodology; project administration; writing – original draft; writing – review and editing.

## FUNDING INFORMATION

None.

## CONFLICT OF INTEREST STATEMENT

None.

## CONSENT

Written informed consent was obtained from the patient to publish this report in accordance with the journal's patient consent policy.

## Data Availability

The data that support the findings of this study are available on request from the corresponding author. The data are not publicly available due to privacy or ethical restrictions.
